# Immunological aspects in chronic lymphocytic leukemia (CLL) development

**DOI:** 10.1007/s00277-012-1460-z

**Published:** 2012-04-12

**Authors:** Ricardo García-Muñoz, Verónica Roldan Galiacho, Luis Llorente

**Affiliations:** 1 Hematology Department, Hospital San Pedro, c/Piqueras 98, Logroño, La Rioja 26006 Spain; 2 Department of Immunology and Rheumatology, Instituto Nacional de Ciencias Medicas y Nutrición Salvador Zubirán, México City, México

**Keywords:** Self-reactive B cells, Receptor editing, Anergy, Clonal deletion, Receptor revision, CD5+ expression, Memory B cell checkpoints

## Abstract

Chronic lymphocytic leukemia (CLL) is unique among B cell malignancies in that the malignant clones can be featured either somatically mutated or unmutated IGVH genes. CLL cells that express unmutated immunoglobulin variable domains likely underwent final development prior to their entry into the germinal center, whereas those that express mutated variable domains likely transited through the germinal center and then underwent final development. Regardless, the cellular origin of CLL remains unknown. The aim of this review is to summarize immunological aspects involved in this process and to provide insights about the complex biology and pathogenesis of this disease. We propose a mechanistic hypothesis to explain the origin of B-CLL clones into our current picture of normal B cell development. In particular, we suggest that unmutated CLL arises from normal B cells with self-reactivity for apoptotic bodies that have undergone receptor editing, CD5 expression, and anergic processes in the bone marrow. Similarly, mutated CLL would arise from cells that, while acquiring self-reactivity for autoantigens—including apoptotic bodies—in germinal centers, are also still subject to tolerization mechanisms, including receptor editing and anergy. We believe that CLL is a proliferation of B lymphocytes selected during clonal expansion through multiple encounters with (auto)antigens, despite the fact that they differ in their state of activation and maturation. Autoantigens and microbial pathogens activate BCR signaling and promote tolerogenic mechanisms such as receptor editing/revision, anergy, CD5+ expression, and somatic hypermutation in CLL B cells. The result of these tolerogenic mechanisms is the survival of CLL B cell clones with similar surface markers and homogeneous gene expression signatures. We suggest that both immunophenotypic surface markers and homogenous gene expression might represent the evidence of several attempts to re-educate self-reactive B cells.

## Introduction

Chronic lymphocytic leukemia (CLL) includes cases featuring somatically mutated or unmutated immunoglobulin heavy variable genes (IGVH). The former consist of cells that likely transited through the germinal center and then underwent final transformation, while the latter consist of cells that likely underwent final transformation prior to their entry into the germinal center. Be as it may, the cellular origin of CLL remains unknown. However, recent advances in our knowledge of CLL biology suggest that CLL results from a proliferation of B lymphocytes selected during clonal expansion through multiple encounters with (auto) antigens, despite the fact that they differ in their state of activation and maturation. The aim of this review is to summarize immunological aspects involved in this process and to provide insights about the complex biology and pathogenesis of this disease. As such, we will discuss the potential harmful steps during the development of lymphocytes, as well as tolerance checkpoints (anergy, deletion, germinal centre exclusion, receptor editing and revision, memory checkpoints, and somatic hypermutation) and immune responses that induce the development and proliferation of neoplastic lymphocytes.

We propose a mechanistic hypothesis to explain the origin of B-CLL clones into our current picture of normal B cell development. In particular, we suggest that unmutated CLL arises from normal B cells with self-reactivity for apoptotic bodies that have undergone receptor editing, CD5 expression and anergic processes in the bone marrow. Similarly, mutated CLL would arise from cells that while acquiring self-reactivity for autoantigens—including apoptotic bodies—in germinal centers are also still subject to tolerization mechanisms, including receptor editing and anergy.

## Definition

CLL is the most common form of leukemia in adults and presents with progressive accumulation of B cells in the blood, bone marrow, and lymphatic tissue. When the disease involves the peripheral blood and bone marrow, it is called CLL, while when lymph nodes or other tissues are infiltrated by cells with identical morphologic and immunophenotypic features to CLL, and yet leukemic manifestations of the disease are absent, it is called small lymphocytic lymphoma. In the World Health Organization classification, the two entities are now considered simply as different clinical manifestations of the same disease.

## Diagnosis of CLL

The diagnosis of CLL requires the presence of at least 5,000 B lymphocytes per microliter in the peripheral blood [1]. Flow cytometry studies performed in patients with leukemic cells include kappa/lambda ratio to assess clonality. The defining feature of the B-CLL clone is the co-expression of CD19, CD20, CD5, and CD23. The levels of surface immunoglobulin, CD20, and CD79 are characteristically low compared to those found on normal B cells [2]. Bone marrow involvement is typically pronounced, with more than 30 % of the nucleated cells in the aspirate being of lymphoid origin.

## Prognostic markers in CLL

Several prognostic factors are currently used for risk assessment prior to the beginning of standard treatment in CLL. For instance, about 50 % of patients with CLL present leukemic cells with somatic hypermutation in rearranged immunoglobulin heavy-chain variable region genes, and they tend to have a more favorable outcome than the other half. The definition of mutated or unmutated CLL is based on an arbitrarily defined threshold of 98 % homology to the most similar germline gene. Overall, the high-risk phenotype is typically associated to unmutated immunoglobulin heavy variable genes [3], expression of the CD38 surface marker [4] and the zeta-associated protein 70 (ZAP-70) [5], as well as to chromosomal aberrations such as 17p (the site of tumor protein p53) or 11q23 deletions (the site of ataxia telangiectasia mutated ATM) [6]. Low-risk phenotype habitually features mutated IGVH, lacks CD38 and ZAP-70, and displays a normal karyotipe or 13q14 deletion. Additional adverse predictive factors include advanced Rai [7] and Binet clinical stage [8], usage of VH3-21 independently on the VH mutation status [9], and short lymphocyte doubling time [10]. These factors predict differences in time to disease progression, time to first treatment, and response to therapy [11].

## Development of B cell repertoire

B cell development occurs initially in the bone marrow and subsequently in lymphoid organs. Later, terminal B cell differentiation also takes place in the bone marrow. In the first step of the whole process, hematopoietic progenitor cells (HSC) differentiate into the earliest identifiable cell type committed to the B cell lineage, the pro-B cell. Interestingly, HSCs of patients with CLL have an increased capacity for generating large numbers of pro-B cells, which undergo clonal selection and ultimately lead to a monoclonal B cell lymphocytosis and then to chronic lymphocytic leukemia B cells [12]. The pro-B cell undergoes a rearrangement of its immunoglobulin (Ig) heavy-chain genes, features cytoplasmic μ chains, and is now called pre-B cell. Subsequent rearrangement of the light chain enables the cell to express surface IgM, thereby becoming an immature B lymphocyte. These cells leave the bone marrow and, on entering the peripheral blood, start to express surface IgD, being now called naïve B cells. They are arrested in the G0 phase of the cell cycle and enter the lymphoid tissue, where they are exposed to antigen-presenting cells, become activated, and differentiate into plasma cells or memory B cells. The memory B cells travel from the extra-follicular area of the lymph node to the primary follicles, where they are confronted with an antigen presented by follicular dendritic cells, resulting in the triggering of a secondary immune response. At this stage, primary follicles change into secondary follicles containing germinal centers. Through activation by an antigen, memory B cells differentiate into centroblasts, resulting in Ig isotype switching and somatic mutations in the variable region of the Ig with the generation of high-affinity antibodies. Centroblasts then progress to the centrocyte stage and re-express surface Ig. The centrocytes with high-affinity antibodies differentiate into either memory B cells or plasmablasts, which subsequently move to the bone marrow and terminally differentiate into plasma cells.

Intriguingly, it is not certain at what stage in lymphocyte maturation CLL arises, since roughly equal numbers seem to originate from a pre- and post-germinal center B lymphocyte [3]. Analyses of immunoglobulin genes and gene expression patterns have defined at least two types of CLL that differ in their tendency towards disease progression: one arises from relatively less differentiated (immature?) B cells with unmutated heavy-chain genes and has a poor prognosis; the other evolves from more differentiated (memory type) B cells with somatically mutated heavy-chain genes and has a good prognosis [13].

## B cell tolerance checkpoints

Tolerance can be mediated by mechanisms that operate at multiple checkpoints throughout B cell development, including clonal anergy, clonal deletion, and receptor editing [14–16]. At an immature B cell developmental stage, B cell receptor (BCR) signaling mediates a remarkable diversity of cellular responses, including developmental progression or arrest, change in location within peripheral lymphoid tissues, survival or apoptosis, and proliferation. During the course of ontogenesis, B cells mature in the bone marrow according to the evolution of Ig chain synthesis. Starting with the rearrangement of VDJ genes for the heavy chain at the pre-B stage, the recombination process continues through the VJ gene rearrangements for kappa or lambda light chain at the immature stage. Thus, the resulting BCR, comprised of randomly selected heavy and light chains, has an unpredictable specificity that could include the ability to “self” bind. If an immature B cell in the bone marrow is not self-reactive, it can leave the bone marrow and transit to the periphery and spleen, where it is called transitional B cell [17]. B cells with reactivity to self-antigens generate moderate to high levels of BCR signaling, which induces biological responses promoting immunological tolerance in the B cell compartment. If an immature B cell binds well to self-antigens, then the resulting BCR signaling causes maturation arrest; however, self-reactive B cells may evade death by switching their heavy- or light-chain expression so as to avoid self-reactivity, a process called receptor editing [18–21]. Self-reactive transitional cells begin the receptor editing process like immature B cells in the bone marrow, but die due to the lack of protective signals provided to immature B cells by bone marrow stromal cells [22]. Similar to immature B cells, CLL cells need survival signals provided by nurse-like cells, cytokines or T cell-related molecules to elude apoptosis [23–25].

It is important to note that autoreactive B cells may undergo receptor editing and anergy in the bone marrow. At the same time, recent evidence shows that light-chain receptor editing occurs not only in bone marrow to cells with a pre-B/immature B cell phenotype, but also in immature/transitional splenic B cells. Nevertheless, editing at the heavy-chain locus appears to occur exclusively in bone marrow cells with a pro-B phenotype [21].

Notably, CLL B cells express CD5 as a surface marker. CD5 cells have been described as a safeguard against autoimmunity and a shield for cancer cells [26].

The specific role of CD5 and IL-10 in protection from autoimmunity has been demonstrated in vivo. The HEL/anti-HEL model is widely used to assess B cell tolerance. In these mice, most B cells express a transgene encoding a receptor for the T-dependent antigen hen egg lysozyme (HEL). Since HEL is artificially expressed in these mice, it is seen as a self-antigen. B cells thus meet their cognate antigen during development, yet fail to be deleted and instead become anergic to HEL and express CD5 [27]. The role of CD5 in the induction of anergy was demonstrated in an elegant experiment where double (HEL and BCR) transgenic mice were bred into a CD5-null background [28]. By contrast to CD5+ animals, these mice developed hemolytic anemia and responded strongly to HEL [28]. This demonstrates that CD5 is necessary to maintain anergy in B cells, thereby inducing self-tolerance. Interestingly, in double-transgenic mice models responding to a T-dependent antigen, HEL and its specific BCR, B cells express CD5 as a result of repeated encounters with their own antigen [27].

Despite most CD5+CD19+ B cells are naïve and represent either transitional B cells or B1 cells that are able to respond to T-independent antigens, a sizeable fraction (up to 25 %) of CD5+ B cells in the blood co-express the memory-associated molecule CD27 [29, 30] suggesting that CD5 is an activation marker. Importantly, CD5 could be induced on B cells in vitro*.* The optimal activating conditions require simultaneous stimulation of both BCR and CD40 surface molecules [29, 31, 32], although IL-6 [31] and the polyclonal activator *Staphylococcus aureus* Cowan Strain (SAC) [33] also stimulate CD5 expression on B cells. For these reasons, CD5 is a marker of some T1/B1 B cells but can also be induced on B2 B cells, indirectly supporting the idea that the origin of CD5+ leukemic B cells could be from self-reactive B cells and not a lineage-specific B cell.

Essentially, CD5 expression maintains tolerance in anergic B cells [28], inhibits early BCR signaling events [34], induces IL-10 secretion in B cells [29] and is associated with receptor editing/revision outside germinal centers [35]. Notably, activation of CD5-negative naïve mature B cells by anti-IgM plus CD40 induces expression of CD5 on a subset of cells, and leads to the upregulation of RAG1 and RAG2 only in cells turned positive for CD5 [35]. This piece of evidence, together with the fact that receptor editing/revision attempts to avoid autoimmunity, suggests that auto-reactive B cells could express CD5+ when their BCRs recognize auto-antigens.

There are also data showing that regulatory checkpoints exist for B cells in the periphery of the germinal center and at the late stages of B cell differentiation into memory or long-lived plasma cells [36, 37]. Germinal center exclusion of self-reactive B cells (9G4 B cells) that express self-reactive antibodies encoded by the IGVH 4-34 gene is an important peripheral checkpoint to avoid the interaction of autoreactive B and T cells, with the subsequent generation of autoantibodies. For this reason, 9G4 B cells only account for 5–10 % of the naïve B cell repertoire in healthy donors, as well as in the IgM memory compartment, and these cells can be found in less than 1 % of germinal centers in tonsil biopsies [36, 38]. Preventing the generation of self-reactive memory B or long-lived plasma cells is another important peripheral checkpoint to avoid autoimmunity. B cells expressing self-reactive and broadly bacterially-reactive antibodies are continuously removed from the repertoire in the transition from naïve to IgM memory B cells, and selection against self-reactive antibodies is implemented before the onset of somatic hypermutation [39].

## Cellular origin of CLL

As mentioned before, CLL cells that express unmutated immunoglobulin variable domains are those that likely underwent final development prior to their entry into the germinal center, whereas those that express mutated variable domains likely transited the germinal center first and then underwent final transformation. Regardless, the cellular origin of CLL remains unknown.

1. Marginal zone B cells as the origin of CLL
  Chiorazzi and Ferrarini suggest that CLL derives from competent B lymphocytes selected for clonal expansion and eventual transformation by multiple encounters and responses to (auto)antigen(s). The observation that the CLL cell surface-marker phenotype (CD5+CD23+CD27+low Igs) does not resemble that of any known normal B cell and that both mutated and unmutated cases show homogenous gene expression signatures with only minor differences [40, 41] has generated a unifying, parsimonious theory according to which CLL clones with either mutated or unmutated IGVHs derive from marginal zone B cells [42]. The authors take advantage of features of marginal zone B cells to explain the origin of CLL. Marginal zone B cells can respond to T-independent as well as T-dependent antigens [43], can display activated membrane phenotypes [44–46] after antigens encounter, and express B cell receptors by unmutated and mutated IGV genes [47, 48]. Interestingly, marginal zone B cells are now seen as major players at the interface between the initial innate and the delayed adaptive immune response [49].
  A typical feature of marginal zone B cells is their capacity to respond to polysaccharide antigens [50]. The ability of marginal zone B cells to respond rapidly and encapsulate bacteria by differentiating into antigen-specific plasma cells helps keeping such infections under control.
  However, the main arguments against a marginal zone origin of CLL are:
  - differences in cell surface phenotype [42],
  - the fact that marginal zone lymphomas usually express *VH1-2* [51] (but biases in VH1-69 and VH4-34 have also been reported) [52],
  - and that even in stage 0, patients with CLL have very poor responses to vaccines. Protein vaccines have produced weak-to-moderate responses in up to 50 % of patients, mainly in early-stage disease with normal serum Ig levels, but responses to polysaccharide vaccines have been virtually zero.
    Importantly, marginal zone lymphocytes are a heterogeneous population of B cells. Despite this, it is possible that some cases of CLL derive from marginal zone B cells that up-regulate CD5 expression after an encounter with either a self-antigen or a super-antigen [33] and escape both central and peripheral tolerance by several mechanisms that include mutations in MYD88 [53, 54].
2. Human B1 cells
  In a very recent article, Griffin et al. [55] have identified the phenotype of human B1 cells. Surprisingly, several similarities between normal human B1 cell and the unmutated B cell subset have been discussed. Both are CD20+CD27+CD43+CD70−, most normal B1 cells express CD5, as do malignant CLL cells, and both express ZAP-70 and ILT3 [55]. The authors suggest that the chronically-activated phenotype of normal B1 cells may predispose to malignant transformation [55]. An argument against human B1 cells as the origin of CLL is the fact that their CDR3 length is inferior to that observed in CLL B. Moreover, B1 cells use different IGHV from those observed in CLL B cells. However, it is possible that during their development, some B1 cells progress to leukemic B cells and, as such, may represent the origin of unmutated CLL B cells [55].
3. Transitional B cells
  B cells that leave the bone marrow need to reach the splenic environment in order to complete their maturation process. Immigrant maturing B cells pass through two transitional stages, known as transitional stage 1 (T1) and 2 (T2). Only a minority of these cells will successfully complete the transition, as this differentiation step is a crucial checkpoint for controlling self-reactivity. Passage through this checkpoint requires the interaction of soluble B cell-activating factor, a member of the tumor necrosis factor family (BAFF), with its receptor BAFF-R, which is primarily expressed on B cells [56].
  Chiorazzi and Ferrarini argue against transitional B cells as the single source of CLL because these cells express CD10, which is not to found on CLL cells unless they have undergone apoptosis. However, the absence of CD27, which is consistently found in CLL, the lack of responsiveness to BAFF/BlyS/TALL-1 [42], the recent discovery of mutations in MDY88 in CLL patients [53], and their role in tolerance in CD19+CD10+IgM+CD27- new emigrant B cells [54] raise the possibility that transitional cells could be the origin of CLL B cells in some cases.

## Pathogenesis and biology of CLL B cells

Although CLL is caused by the accumulation of neoplastic lymphocytes in the G0 phase of the cell cycle, some studies have demonstrated that a proportion of neoplastic CLL lymphocytes, ranging between 0.1 and 1 % per day, are actively duplicating. Furthermore, patients with proliferation rates greater than 0.35 % per day have been found to have a more aggressive disease [57, 58]. The analysis of lymphoid tissues involved in CLL has revealed focal, scattered aggregates of large, proliferating leukemic cells that form nodular areas in lymph nodes and bone marrow [23, 59]. Numerous CD4+CD40L+T cell infiltrates are found in close contact with the proliferating CLL cells [60], and micro-environmental interactions with bone marrow stromal cells are able to extend the survival of CLL upon direct contact [59]. Thus, the CLL population may originate from a clone with few or no V- domain mutations, or from a more mature clone whose V domains have undergone the hypermutation process. This creates two separate pools of B cells, both of which originate from antigen-stimulated B lymphocytes.

CLL is now viewed as a proliferation of B lymphocytes selected by clonal expansion through multiple encounters with (auto) antigens, despite the fact that they differ in their state of activation and maturation. However, the fact that both mutated and unmutated CLL B cells recognize autoantigens [61, 62], differing from each other by relatively few expressed genes [41], and have stigmas suggesting that they have been re-educated to avoid self-reactivity [29, 61, 63–76], suggests that CLL is originated by a coordinated normal immunologic tolerance mechanism to destroy self-reactive B cells. For these reasons, we believe that CLL B cells are made, not born.

What is the role of central tolerance mechanisms in the promotion of CLL B cell development? In an elegant series of papers, Nemazee and coworkers have demonstrated that two mechanisms of immune tolerance are used within the bone marrow when immature B cells encounter self-antigens and that the mechanism employed depends on the state of maturation of the B cell. The first mechanism used to confer B cell tolerance is referred to as receptor editing. If an immature, self-reactive B cell encounters a self-antigen in the bone marrow, the process promotes rearrangement of the second immunoglobulin receptor light chain in the hope of altering BCR specificity. In one study, two thirds of autoreactive immature B cells were found to undergo receptor editing without any significant apoptosis [77]. Another study found that later in B cell development, that is after receptor editing has occurred, immature B cell engagement with self-antigens leads to apoptosis within the bone marrow [78]. Intriguingly, receptor editing and receptor revision may have undesirable consequences. Once a heavy chain contributes in a dominant fashion to self-reactivity, a switch in an antibody’s light chain may not fully abrogate autoreactivity. For this reason, receptor editing does not necessarily result in the correction of autoreactivity, and might influence the formation of polyreactive B cell receptors [79–81]. In addition, the edited antibody will usually have a longer CDR3, which in itself may predispose it to autoreactivity or polyreactivity [79]. This could explain the greater level of polyreactivity and autoreactivity of unmutated CLL cells, as well as the reason for antibodies produced from mutated CLL rearrangements can display polyreactivity after their IGHVs are reverted to the germline sequence [61, 82].

### Evidence of receptor editing in CLL B cells

The information relating to the receptor editing process, which is involved in the control of self-reactive CLL B cells that recognize autoantigens [61] and secrete autoantibodies under stimulation [83], is supported by the following observations:

- The significant role of immunoglobulin light chains in antigen recognition and selection in CLL [64]. This observation suggests that CLL cells are in active, (auto)antigen-driven receptor editing process.
- Both CLL subsets can express unusually long HCDR.[65] This supports that CLL cells have edited receptors.
- A subset of CLL patients have leukemic B cells that express more than one functional Ig heavy chain [66], suggesting that this lack of allelic exclusion could be related to the receptor editing/revision process.

The fact that stereotypical CLL BCRs recognize autoantigens in stromal cells [84] is an example of a situation where receptor editing could be in a position to try to avoid autoimmunity in a definite microenvironment. Interestingly, 79.3 % of unmutated CLL antibodies are polyreactive [61], and reactivity with a particular form of apoptotic cells is a common feature of this subset [62]. Intriguingly, the unmutated CLL subset expresses antibodies with long heavy and light-chain CDR3 [61]. Moreover, some cases of CLL have multiple light-chain rearrangements [64], a feature that could imply receptor editing/revision and advocate that CLL leukemic B cells undergo receptor editing to avoid autoimmunity.

Some illustrative examples of receptor editing in CLL include a number of cases that express lambda light chains with either potentially functional *IGKV1-17* gene rearrangement [64], which has been associated with the cationic charge of autoantibodies in lupus nephritis [85], or stereotyped *IGHV3-21* CLL cases, which are characterized by a strikingly biased expression of lambda light chains [64, 86, 87]. In addition, lambda chains are more efficient at silencing autoreactive/polyreactive antibodies than kappa chains in humans [43]. Importantly, the light-chain rearrangements of *IGHV3-21* CLL cells have followed the hierarchical pattern of light-chain recombination (IgK, IgK, and IgL) and have undergone several attempts of rearrangement before producing a functional light chain [64, 86, 87].

At this point in the discussion, we believe that subset receptor editing fails to abrogate autoreactivity or polyreactivity in the unmutated CLL; however, in some mutated CLL subsets, receptor editing/revision has successfully worked to avoid self-reactive BCRs.

Despite these arguments, the current hypothesis is that mutated cases avoid autoreactivity by somatic mutations, not by receptor editing mechanisms. This is supported by the observation that biased mutations in the heavy and light chains could diminish the responsiveness of CLL progenitors to autoantigens [88]. The *IGHV4-34/IGKV2-30* CLL stereotype is characterized by “long” positively-charged HCDR3 enriched in aromatic and positively-charged amino acids, similar to pathogenic anti-DNA antibodies.[89] However, the fact that *IGHV4-34* sequences should undergo somatic hypermutation in order to negate their autoreactivity, and that the introduction of even a few strategically positioned aspartic acid residues in the kappa chains of the transgenic animals may be sufficient to abrogate DNA binding by 3 H9 anti-DNA autoantibodies, supports the idea that a number of mutated cases avoid autoreactivity by somatic mutations [67, 80, 89, 90]. Even so, in the periphery, the expressed light chain may also be inactivated by somatic mutation, and an autoreactive light chain can be re-expressed [91]. It is even possible that an editing event may occur in peripheral mature B cells, and may produce de novo autoreactive specificity. Fascinatingly, in five of six CLL samples lacking Ig heavy-chain allelic exclusion, approximately one of the two heavy-chain rearrangements could be considered unmutated [66]. Conceivably, these CLL samples undergo an editing in the heavy chain to avoid self-reactivity. Moreover, the authors suggest that the allele with somatic mutations had undergone rearrangement first and was expressed by the B cell clone during an antigen-driven immune response [66]. Bias in the usage of the VH3-21 gene has been reported in both unmutated and mutated CLL B cells, often in combination with lambda light chains and selected variable lambda genes [92]. Interestingly, “one mutated *VH3-21-*using case displayed double rearrangements with one functional *VH4-61* gene rearrangement but a nonfunctional *VH3-21* gene rearrangement with a stop codon introduced in the CDR3” [92]. This indicates that if their original Ig receptor develops enhanced binding activity for self-antigens through somatic mutation [93, 94], the B cell might rearrange the other heavy-chain allele to avoid autoreactivity.

This suggests that mutated CLL B cells might arise because of somatic mutation during germinal center reactions, and try to avoid autoreactivity through a receptor editing/revision process. For these reasons, it is possible that autoreactivity could be avoided in mutated cases by receptor editing/revision and not only by somatic mutations.

In summary, it is possible that CLL cells avoid the initial machinery of central tolerance and, despite undergoing a receptor editing process, both mutated and unmutated CLL cells maintain a self-reactive BCR and progress to transitional and mature B cells [12, 22]. These details support the concept proposed by Chiorazzi, according to which CLL is a clonal disease of B lymphocytes with receptors that vary in specificity for autoantigens [95].

Anergy is another important mechanism to maintain tolerance. There are many self-antigens that are not encountered by the developing B cell population or that do not have the capacity to cross-link BCRs to a sufficient degree to elicit the receptor editing/clonal elimination process. Such cells, even when mature, may nonetheless be inactivated through a process that involves the cross-linking of receptors without the reception of critical costimulatory signals. These inactivated cells may be retained in the body but are unresponsive to antigens and are referred to as anergic. Importantly, when removed from the presence of the anergy-inducing stimulus, anergic cells may regain responsiveness. Notably, an anergic B cell population has been identified in humans.[96] The CLL cells characteristically express low levels of surface Ig, and this fact has been considered to be an indication that the leukemic cell has been anergised, probably by exposure to antigens [68, 69]. Remarkably, repetitive BCR stimulation of normal B cells leads to anergy and CD5 expression, both of which are features of CLL.

Importantly, anergy could diminish the lifespan of anergised B cells if they do not receive other survival signals [97–99]. It is interesting to note that the impairment of anergic B cell activation appears to be at the level of BCR signaling, because the activation of proliferation through either CD40 or TLR is unaffected in the absence of antigens.

Clonal anergy could be involved in both central tolerance in the bone marrow and peripheral tolerance in the spleen and lymph nodes. For this reason, we believe that it could be involved in both mutated and unmutated CLL subsets. Muzio et al. showed that CLL B cells that do not respond to BCR ligation (typically belonging to the mutated CLL subset) show activation of cellular pathways which suggests anergy [70]. In general, BCR ligation in the unmutated CLL subset leads to predominantly activating and proliferative responses, whereas BCR signaling in the mutated CLL subset favors anergic and antiapoptotic responses [63, 100, 101]. Intriguingly, the differences in BCR responses in both subsets might be influenced by disturbances in CD5 expression and function. CD5 protects from autoimmunity by several mechanisms that include the inhibition of BCR early signaling events [34], maintains tolerance in anergic B cells [28], and induces the secretion of IL-10 in B cells [29]. Unfortunately, in some cases CD5 does not properly inhibit BCR-mediated signaling in CLL B cells [71]. Disturbances in CD5+ function have been detected in subsets of patients with CLL. Significantly, CD5 does not regulate the signaling triggered through BCR in B cells from a subset of B-CLL patients [71], provides viability signals to B-CLL cells [72, 73], promotes IL-10 secretion in CLL-B cells [74], and protects from apoptosis [75].

Importantly, sustained or repetitive BCR signaling promotes survival in CLL cells [102, 103]. A significant correlation has been reported between the polyreactivity of the BCR and an aggressive clinical course of the disease [76]. This aggressive clinical course was even more pronounced when patients with CLL had BCRs that recognized ≥5 epitopes (“multireactive BCRs”), suggesting that BCRs that react with various epitopes may be more prone to sustained signaling [76].

Experimental evidence, combined with clinical observations such as the fact that the transformation of CLL to large cell lymphoma (Richter’s Syndrome) and the progression toward a more malignant phase of the disease characterized by an increased number of prolymphocytes is often associated with immunophenotypic drift where CD5 is lost, supports the notion that CD5 is important in CLL development and proliferation.

The fact that unmutated and mutated CLL cells derive from self-reactive precursors [61], the suggestion that CLL cells are in active (auto)antigen-driven receptor editing [64], autocrine IL-10 secretion during CD5+ stimulation in leukemic CLL B cells [72, 74], and that most if not all cases of CLL involve the production of polyreactive monoclonal antibodies that react with several types of autoantigens, support the idea that CD5 expression in CLL might be related to the control of autoimmunity. For these reasons, we believe that CLL leukemic cells take advantage of impaired anergy, CD5 expression and IL-10 secretion in some cases. In summary, anergy could be important to maintain a more benign disease in mutated CLL B cells; however, the unmutated subset appears to be more resistant to this tolerance mechanism.

As a final point, in vivo multiple structurally divergent antigens can bind and stimulate CLL B cells through the BCR. This fact suggests that these cells could feature a cross-reactive BCR that recognizes both self- and foreign antigens generated during an immune response against foreign antigens. This could explain the fact that mutated non-autoreactive CLL antibody sequences reverted in vitro to their germline counterparts encode polyreactive and autoreactive antibodies [61]. In addition, not all autoantigen-binding BCRs are necessarily detrimental to the organism. A proportion of BCRs may bind autoantigens with too low an affinity to trigger an autoimmune response, but may bind strongly enough to invading pathogens to exert a protective host-defense effect. Importantly, several BCRs in CLL clones that bind apoptotic cells and self-structures also bind epitopes expressed on the coat of common bacteria [95, 104–106]. Some of these CLL clones are from the mutated CLL subset. Interestingly, the development of CLL shortly after bacterial pulmonary infections and some common community-acquired infections has been reported [107, 108]. Current data support the idea that the mutated B cell subset could derive from self-reactive B cells that have lost their self-reactivity when passing through the germinal center reaction by somatic hypermutation [61, 67]. However, some CLL cells expressing mutated or borderline mutated *IGVH* genes could recognize autoantigens [61, 62, 104, 105], thus advocating the fact that self-reactivity or polyreactivity may be acquired during somatic hypermutation or that an autoreactive B cell passes several tolerance checkpoints. Nevertheless, the immune system somehow turns these cells into “anergic-memory B cells” that cannot produce high-affinity autoantibodies. This implies that not all autoreactivity is corrected by somatic hypermutation.

## Hypothesis about the development of CLL B cells through tolerance mechanisms

The observation that mutated and unmutated subsets of CLL have distinct clinical courses is consistent with a two-cell origin model; however, the homogeneous gene expression signature, with only minor differences between unmutated and mutated CLL, suggests a singular originating cell and the one-cell model.

**Fig. 1:**
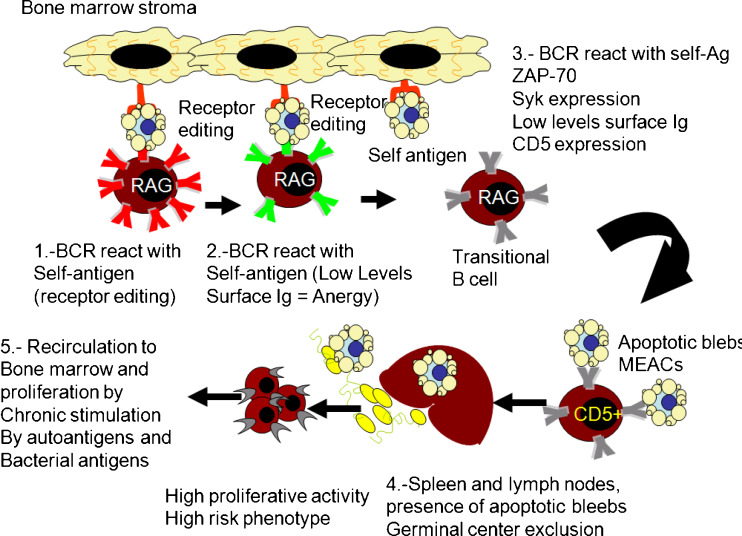
Hypothetical immunologic mechanisms implicated in the unmutated CLL subset. The unmutated CLL subset might derive from any (non-lineage specific) autoreactive B cell that experience several tolerance mechanisms and are chronically under check by the persistence of autoantigens. The cell surface markers and gene expression of CLL cells could simply be the result of several immunologic mechanisms that try to destroy or avoid the persistence of self-reactive B cells; these mechanisms include receptor editing, anergy, clonal deletion, receptor revision, CD5+ expression, germinal center exclusion, and memory B cell check points

**Fig. 2:**
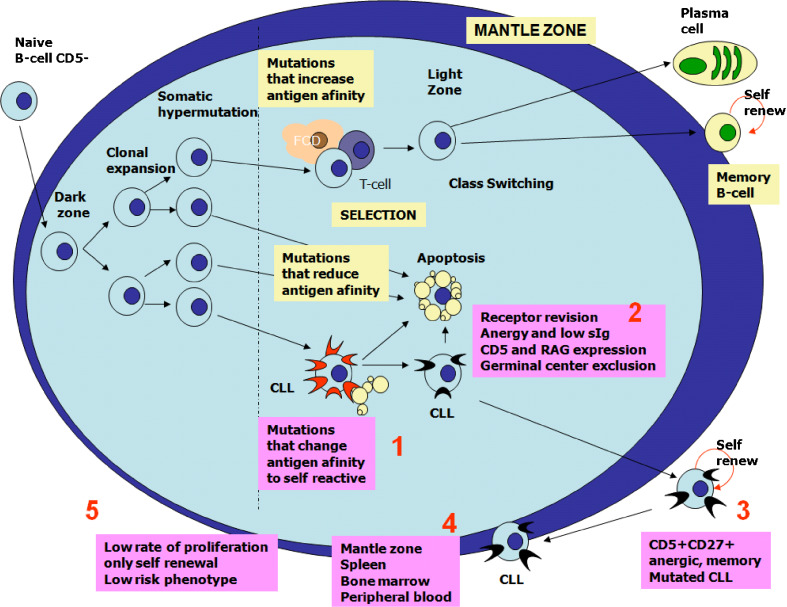
Hypothetical immunologic mechanisms implicated in the mutated CLL subset. The mutated CLL subset might derive from any B cells that acquire “de novo self-reactivity” while undergoing the somatic hypermutation process. Following this “de novo auto-reactivity” development, a normal CD5- B cell can theoretically be transformed into a “de novo auto-reactive” memory B cell that expresses CD5 (increasing the threshold for BCR activation), undergoes receptor revision (changing light chain to evade autoimmunity), down-regulates surface Ig (to avoid activation), and remains under check by germinal center exclusion (to diminish the chance to progress in the maturation and become plasma cell). Finally, all these tolerance mechanisms converts the CD5- B cell into an “anergic-edited, CD5+CD27+ memory B cell” excluded from germinal centres

We propose an alternative theory in that an “original autoreactive B cell” (unmutated) or a “de novo autoreactive B cell” (mutated) undergo the same tolerance mechanisms either in the bone marrow (central) or in the periphery (lymph nodes), leading to homogenous gene expression. On the one hand, in this theory, original autoreactive B cells (unmutated) undergo receptor editing, anergy, and CD5 expression without success, leading to unmutated CLL cells with autoreactivity, retention of signaling through BCRs and poor prognosis (Fig. 1). On the other hand, a normal B cell that acquires autoreactivity during a somatic hypermutation process successfully undergoes receptor revision, anergy, CD5 expression and possible germinal center exclusion, leading to mutated CLL cells without autoreactivity or anergy, and only with self-renewing, low/absent BCR signaling and good prognosis (Fig. 2). Variations of this theory have been proposed to explain other CD5+ B cell lymphomas [109].

All in all, we suggest that rather than having a cellular origin or cellular subtype like marginal zone B cells [42], CLL is originated by a coordinated normal immunologic tolerance mechanism to destroy self-reactive B cells and to avoid autoimmunity. This hypothesis is supported by several lines of experimental evidence. CLL development might be influenced by autoantigen recognition in both mutated and unmutated subsets, and IGVH gene usage is often associated with autoantibody reactivity [42, 61, 65, 83].

Intriguingly, receptor editing and receptor revision may have undesirable consequences. For instance, receptor editing may not always correct autoreactivity, and in the case of VH replacement, the retention of much of the CDR3 may preserve some of the autoreactivity. In addition, VH replacement antibodies will usually have longer CDR3, which in itself may predispose them to auto- or poly-reactivity. Likewise, light-chain editing, while more frequent than heavy-chain editing and more likely to occur at a time when BCR specificity is being tested, does not necessarily result in the correction of autoreactivity.

Based on HCDR3 length, both CLL subsets can express unusually long HCDR3 [65], suggesting that CLL cells undergo receptor revision and editing during their development [64]. Importantly, the polyreactive binding activity of natural antibodies may be dependent on somatically-generated CDR3. Moreover, despite the expression of the CD27 surface marker, unmutated Ig sequences have been observed in healthy and rheumatoid arthritis patients, suggesting that these cells may still be under a diversification process [110]. We speculate that this diversification process could indeed consist in receptor editing or revision to avoid autoimmunity. Importantly, this could at least in part explain a proportion of unmutated, CD5+ CLL clones with unusually long HCDR3 that express CD27 as well, a “marker of marginal zone B cells, and memory B cells”.

Anergic B cells are characterized by chronic, low-level BCR signaling. They also exhibit reduced surface IgM levels, but can express high levels of IgD. The low expression of BCR is the hallmark of CLL and anergic B cells. Interestingly, an in vitro analysis of signal competence reveals that unmutated CLL cells generally continue to respond, whereas mutated CLL cells are anergised [63]. CD5 expression protects B lymphocytes from uncontrolled self-reactivity by increasing the BCR signaling threshold; however, CD5 does not properly inhibit BCR-mediated signaling in CLL B cells [72]. In addition, CD5+ can also promote the autocrine production of IL-10 [71, 72]. Interestingly, these mechanisms could promote the release of regulatory IL-10 [111], thereby fostering the generation of B cells [69], with the subsequent capacity to induce an infectious tolerance and block T cell immune responses [112].

Importantly, mechanisms meant to increase diversity and tolerance, like somatic hypermutation or receptor editing/revision, respectively, could generate double-stranded DNA breaks, and DNA repair enzymes could, in theory, generate both translocations and deletions. It is possible that an autoreactive cell that repeatedly goes through tolerance checkpoints develops a clonal evolution with acquisitions of translocations like t(11:14) in mantle cell lymphoma or 13q, 11q, or 17p deletions in CLL.

Additionally, monoclonal B cell lymphocytosis or regulatory B cells [69] could also result from these immunological tolerance mechanisms.

This theory could explain the origin of the cell surface marker phenotype of CLL (CD5+CD23+CD27+low Igs) and suggests that both mutated and unmutated cases show homogenous gene expression signatures, with only minor differences because both subsets undergo the same tolerogenic mechanisms. Differences in the “success” of this tolerance mechanism could be behind the great variety of clinical presentations in patients with CLL.

## Other immunological alterations that might theoretically predispose to the loss of CLL clone control

Deficiency in self-antigen retention induced by defects in complement components or impaired clearance of apoptotic B cells could possibly lead to an increased release of self-reactive B cells from bone marrow to periphery. On the one hand, defects in the complement system might cause a deficient presentation of antigens in the bone marrow and diminish the protection of the receptor editing mechanism. On the other hand, impaired clearance of apoptotic cells in the bone marrow induces an increased stimulation of immature, self-reactive B cells that could undergo sustained receptor editing in the bone marrow or avoid tolerance. Importantly, receptor editing can also produce polyreactive B cells or a simple change in recognition from an autoantigen to another that recognizes the newly-edited BCR in an immature B cell. These two mechanisms could be involved in the generation of mantle cell lymphoma [109] or CLL with unmutated IGHV genes [64].

Patients with CLL have several disturbances in the complement system [113–115]. Abnormalities in C4 could be present at several stages of the disease; vice versa, C1q deficiency is restricted to more advanced stages [115]. Interestingly, abnormalities in the complement system have been associated with predisposition to both infections and autoimmunity [116]. An interesting point of view is that low levels of complement could result in a loss of central tolerance in the bone marrow, leading to the emergence of autoreactive clones and predisposing to autoimmunity [117–119], especially autoimmune haemolytic anemia and immune thrombocytopenia.

It is remarkable that almost nothing is known about the autoantigen-presenting modes that establish central B cell tolerance. However, genetic deficiencies in the complement components C1q and C4 or secreted natural serum IgM lead to systemic autoimmune diseases. Two models have been proposed to explain the emergence of autoantigen-reactive B cells. In one model, macrophages expressing the appropriate complement receptors (C1qR and CR1) efficiently remove apoptotic cells that are bound by natural IgM, C1q and C4b, thereby preventing the accumulation of these cells and subsequent activation of mature B cells [117]. The other model suggests that autoantigens from apoptotic cells are presented to immature B cells by immune complexes containing C1q, C4b, and IgM on a yet unidentified cell expressing the appropriate receptors C1qR, CR1, and possibly FcRμ. In the latter model, this antigen presentation can result in negative selection and/or anergy [120]. A better understanding of the modes of central tolerance induced by apoptosis, editing, anergy and ignorance requires the identification of relevant antigen-presenting cell populations. Despite this, some authorities on CLL suggest that CLL cells themselves “once they undergo apoptosis within infiltrated peripheral tissues” might be involved in self-perpetuating the disease by providing autoantigenic targets [121]. In support of this model, Tripodo et al. discovered C1q production by bone marrow stromal cells, an important part of complement that is involved in the clearance of apoptotic cells [122]. Defects in complement, bone marrow infiltration, the high frequency of self-reactive/polyreactive CLL B cells, and the increased presence of autoimmune phenomena in advanced stages of CLL suggest that it is possible that apoptotic cells in this compartment play an active role in disease progression.

How are complement and immunoglobulin deficiencies related to the processes that lead to CLL development?

Molecular motifs exposed on the surface blebs of apoptotic cells or microbial epitopes expressed on the surface coat of common bacteria may be relevant in triggering and/or facilitating the evolution of CLL [105, 123]. This is important because the impaired clearance of autoantigens or the presence of bacterial antigens that mimic self-structures could contribute to increasing antigen-driven proliferation of CLL B cells.

**Fig. 3:**
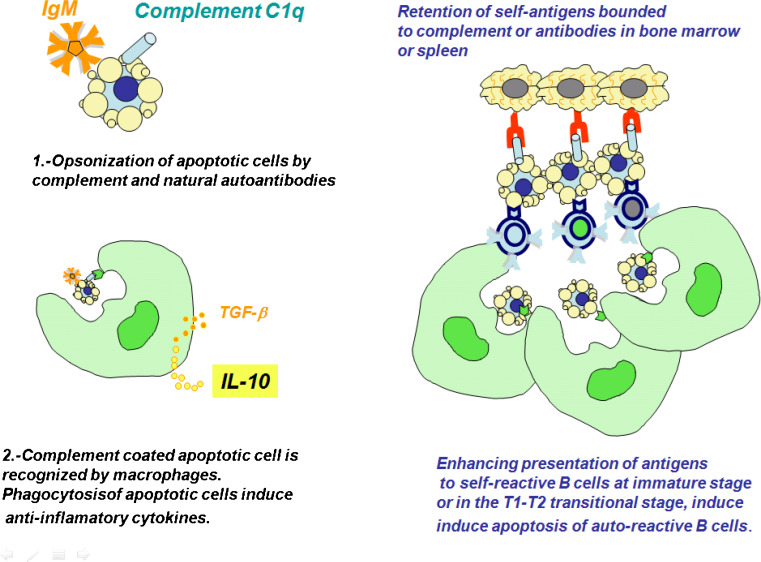
The role of complement in apoptotic cell recognition and prevention of autoimmunity. Complement binding to immune complexes or to auto-antigens protects from autoimmunity by enhancing presentation of antigens to self-reactive B cells at an immature stage. Phagocytosis of apoptotic cells generally induces the release of anti-inflammatory cytokines

Apoptotic cells are recognized by multiple receptors, including complement C3 and C1q fractions, as well as natural autoantibodies (IgM) [124–126]. Natural IgM autoantibodies bind to phospholipids exposed on apoptotic cells and also activate the classical pathway, generating C1q, C4b, C3b, and iC3b ligands for complement receptors. Phagocytosis of apoptotic cells generally induces anti-inflammatory cytokines such as transforming growth factor B and IL-10 [124, 125] (Fig. 3). The association between complement or immunoglobulin deficiencies and CLL could be attributed to a failure of complement-dependent opsonization, resulting in an accumulation of apoptotic cells and a release of autoantigens that constitute a continuous stimulus for CLL clone proliferation (Fig. 3).

An overall reduction in antibody production can also contribute to the inability to eradicate microbial pathogens with structures similar to autoantigens, which leads to chronic stimulation for the CLL clone.

**Fig. 4:**
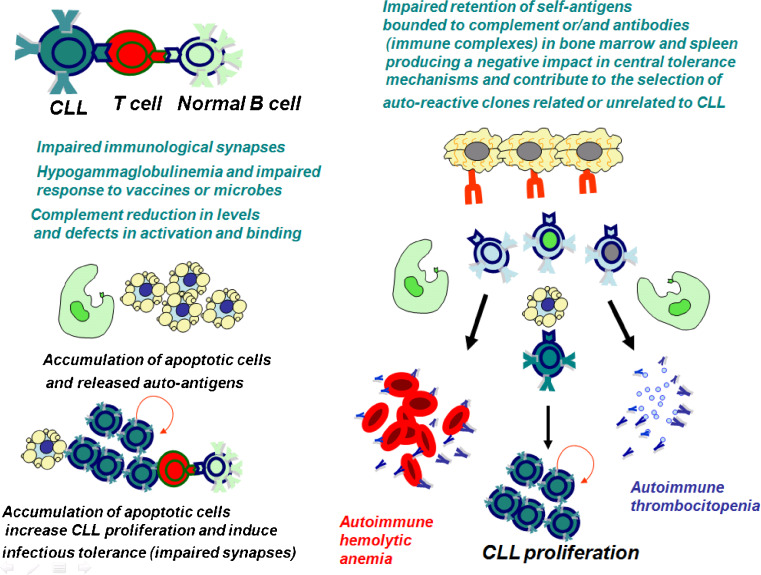
Complement deficiencies and low levels of immunoglobulins observed in CLL could contribute to autoimmunity

Moreover, complement binding to immune complexes or to autoantigens could protect from autoimmunity by enhancing the presentation of antigens to self-reactive B cells at the immature stage or in the T1–T2 transitional stages [127] (Fig. 3). Complement deficiencies and low levels of immunoglobulins observed in CLL could contribute to impaired retention of self-antigens bound to complement and/or antibodies (immune complexes) in the bone marrow and spleen, having a negative impact on central tolerance mechanisms and contributing to the selection of autoreactive clones that are related or unrelated to CLL (Fig. 4).

This could explain why autoantibodies causing autoimmune thrombocytopenia and hemolytic anemia are produced by residual normal B cells in CLL patients. The mechanisms of hematological autoimmunity in CLL could be similar to those observed in common variable immunodeficiency (CVID) patients [128].

## CLL disease-associated immunosuppression

**Fig. 5:**
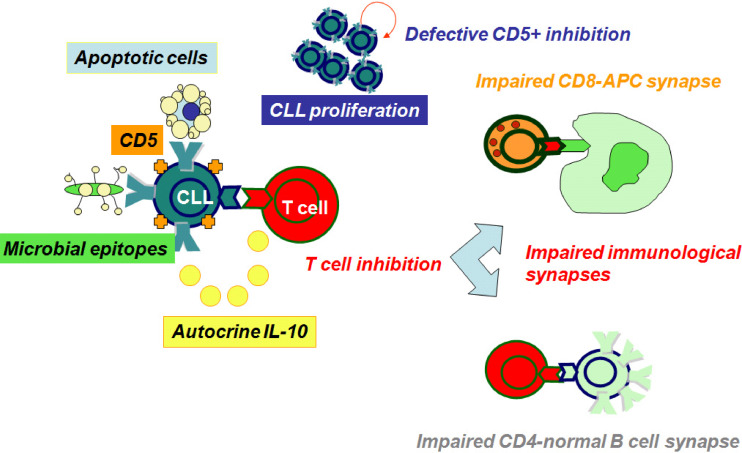
Infectious tolerance induced by CLL-B cells

IL-10 is a potent inducer of B lymphocyte differentiation as well as an inhibitor of T cell lymphocytes and antigen-presenting cells [129]. Autocrine IL-10 is produced by autoreactive (systemic lupus erythematosus SLE) [130, 131] and immature CD5+ B cells [132]. Significantly, IL-10 could protect from apoptosis and induce the expression of BCL-2 in B cells [129]. Notably, CD5 provides viability signals and leads to IL-10 production, which acts as an autocrine growth factor for leukemic B cells [72]. However, CD5 does not properly inhibit BCR-mediated signaling in CLL B cells [71]. It is possible that autoantigen stimulation through BCR in an autoreactive B cell guide the expression of CD5+ by increasing the threshold for BCR activation to avoid the process leading to antibody production. Moreover, it could also induce the autocrine production of IL-10 to down-regulate costimulatory molecules like CD80, thus promoting an immunosuppressive microenvironment that blocks the activation of T and antigen-presenting cells to avoid autoimmunity. In CLL, this mechanism might induce a process known as infectious tolerance [133] in cell–cell contact, mediated by autocrine IL-10, in a similar manner to regulatory T [134] or B cells [135, 136]. If these autoreactive B cells persist and proliferate by autoantigenic stimulus, presumably a persistent T cell inhibition could be maintained in a straight line with the load of autoreactive CD5+ and IL-10+ B cells. This hypothesis could explain the existence of defective synapses in CLL (Fig. 5).

Still, open questions are: then, why and when a B cell expresses CD5 antigen and produces autocrine IL-10 as it comes to recognize an autoantigen. The answer could be that during the tolerance process in bone marrow or during somatic hypermutation, B cells must be disconnected from T cells, since autoimmunity could be corrected (receptor editing) or abrogated (anergy or deletion). The migration of immature T cells from the bone marrow to the thymus reduces the opportunity to interact with immature B cells. In a germinal centre, if a B cell becomes self-reactive, it could be excluded or tolerized in the absence of T cell help (deletion of autoreactive T cells specific for an autoantigen in the thymus). Interestingly, IL-10-producing regulatory B cells are enriched in both memory (CD27+) and transitional (CD38high) B cell compartment [135]. We hypothesize that CLL leukemic B cells could represent a self-reactive (e.g., CD5+ expression and autoreactive-related usage of *IGHV*) regulatory B cell (e.g., production of IL-10, exspression of CD27) clone that is driven to proliferate by the common environment and the autoantigens that they recognize. This mechanism could support the previous suggestion that CLL cells might behave like regulatory B cells [69].

## Conclusion

Stem cells from patients with CLL produce an increased amount of pro-B cells. These pro-B cells undergo tolerance in bone marrow stroma and the peripheral lymphatic system. We suggest that during several stages of B cell development CLL B cell clones might emerge when self-reactive B cells undergo receptor editing/revision, anergy, CD5+ expression and somatic hypermutation. CLL is a proliferation of B lymphocytes selected from clonal expansion through multiple encounters with (auto)antigens, despite the fact that they differ in their state of activation and maturation. Autoantigens and microbial pathogens activate BCR signaling and promote tolerogenic mechanisms such as receptor editing/revision, anergy, CD5+ expression and somatic hypermutation in CLL B cells. These mechanisms support the generation of new poly-reactive or oligo-reactive BCRs in unmutated or mutated CLL B cell subsets, respectively. Autocrine immunosuppressive cytokines produced by CLL B cell clones can help to produce defects in T cells. Survival of CLL B cell clones with similar surface markers and homogeneous gene expression signatures that is with only minor overall differences, might be a sign of several attempts to re-educate self-reactive B cells. It is possible that self-reactive B cells that are repeatedly exposed to tolerance checkpoints develop a clonal evolution with acquisitions of surface markers and deletions like 13q, 11q, or 17p that are associated with CLL.
